# Antiviral usage for H1N1 treatment: pros, cons and an argument for broader prescribing guidelines in the United States

**DOI:** 10.1371/currents.RRN1122

**Published:** 2009-11-11

**Authors:** Edward Goldstein, Marc Lipsitch

**Affiliations:** ^*^Harvard School of Public Health and ^†^Dept of Epidemiology and Center for Communicable Disease Dynamics, Harvard School of Public Health

## Abstract

Current CDC guidelines for antiviral treatment of people with influenza like illness (ILI) effectively discourage treatment of people with no underlying medical conditions unless they exhibit severe symptoms, such as evidence of lower respiratory tract infection or clinical deterioration. This guidance is unlike that provided by some other countries, which allow for treatment of most moderately symptomatic individuals. We examine evidence for benefits of antiviral usage for influenza treatment, including its relation to severe outcomes for the current pandemic H1N1 strain. We also discuss some of the potential cons of antiviral usage. In the current situation in the US, with an elevated and evidently growing burden of influenza hospitalizations and mortality, a high percentage of individuals infected with influenza (with almost all of those carrying the H1N1pdm strain) among those who exhibit ILI and get tested for influenza virus, very low levels of antiviral resistance and little time left for antiviral resistance to take off before large quantities of vaccine become available, we think it is worthwhile to consider a revision to the current antiviral usage recommendations, such that physicians would be encouraged to consider prescribing antivirals to individuals with moderate to severe symptoms who present for treatment.

Note: Very recently CDC has adopted clarifications for its antiviral usage guidelines: http://www.cdc.gov/H1N1flu/antivirals/facts_clinicians.htm

**Benefits of antiviral usage for influenza treatment: **The ability of antiviral drugs to reduce symptoms and shorten their duration are well documented, particularly when antivirals are given early in the course of illness [1],[2],[3].There is evidence that early usage of antivirals for treatment of infected individuals can reduce transmission to others [4],[5],[6]; [4] in particular applies to the current pandemic H1N1pdm strain, though the findings in that study are not statistically significant. A 2003 study suggests a 60% reduction in influenza hospitalization rates for patients who received early antiviral treatment [7].

Data in [8] show that among hospitalized patients, severe outcomes (ICU admission and/or death) were less likely in patients who received antiviral treatment within two days of symptom onset; the same conclusion is true for lethal outcomes for hospitalized patients in http://jama.ama-assn.org/cgi/content/short/302/17/1896?home . We have attempted to extract further information on the effects of antiviral usage on severe outcomes for the current H1N1pdm strain by an ecological comparison between two neighboring Southern Hemisphere countries that have experienced a complete H1N1pdm season: Chile and Argentina.

**Chile vs. Argentina: **Chile had an active antiviral usage policy during the course of the epidemic, with almost 650,000 courses distributed (~ 4 courses for every 100 persons in a country of 17 million) by August 16 [9]. The current number of confirmed fatalities in Chile is 136, i.e. 0.8 deaths per 100,000 inhabitants [10].  In Argentina, treatment was reserved for hospitalized patients until after the peak of the epidemic, at which point policy changed to include treatment of pregnant women and members of high risk groups; only near the end of the epidemic was treatment of all ILI cases recommended ([11], p. 25). Argentina had 580 confirmed fatalities in a population of 40.4 million [12], or 1.44 deaths per 100,000 inhabitants.

One might argue that the difference could be due to better ascertainment of fatalities in Argentina.  However, overall ascertainment of cases appears to be higher in Chile than in Argentina: Chile has 12,257 confirmed cases and 136 confirmed fatalities [10], while Argentina has 9,119 confirmed cases and 580 confirmed fatalities [12].  It is thus likely that a higher percentage of H1N1-realted deaths were ascertained in Chile than in Argentina.

A second line of ecological evidence for the benefits of early antiviral treatment appears within the Argentinean data in [11], p. 18, in which the percentage of fatalities among the confirmed H1N1 infections in pregnant women has dropped significantly after guidelines for treatment of high risk individuals were adopted. More precisely, the data shows 174 pregnant women with confirmed H1N1 infection with symptom onset prior to the adoption of those guidelines; 31 of them (17.8%) died. Among the 69 pregnant women with confirmed H1N1 infection with symptom onset after those guidelines were adopted, there were 4 fatalities (5.8%). The difference is statistically significant (Fisher’s exact test p-value =.015; 95% CI for the odds ratio of a pregnant woman’s death following infection after the guidelines adoption vs. prior to the guidelines adoption is (0.07, 0.85)).

**High vs. low risk individuals: **In [13] we have argued for pre-dispensing antivirals to high risk individuals, aiming to initiate treatment early, to reduce their risk of progression to severe disease. On Sep. 8, 2009 CDC has revised its guidelines for antiviral usage (subsequently updating those on Sep. 22 and Oct. 16), further encouraging early treatment of individuals with a high risk of complications, and indicating that treatment should not wait for laboratory confirmation of influenza [14].  At the same time, those recommendations state [14]:

“Most healthy persons who develop an illness consistent with uncomplicated influenza, or persons who appear to be recovering from influenza, do not need antiviral medications for treatment or prophylaxis.”

This has, in effect, discouraged antiviral treatment for many individuals.  This is a lost opportunity to reduce severe morbidity among “low-risk” individuals, who make up a sizeable percentage of the severe cases (hospitalizations and fatalities), though exact percentages vary between 15%-50% in different studies [15],[8],[10],[16],[17],[18]. We are not aware of data assessing this lost opportunity so far, save for some anecdotal evidence [19].

**Current situation in the US and an argument for policy change:** CDC data up to week 41 (ending October 17) suggests a rise in all indicators related to the epidemic’s progression in the US, including hospitalizations and confirmed fatalities [20]. Pneumonia and influenza mortality rates in the 122 city reporting system have been above the epidemic threshold for 3 consecutive weeks [20]. While not all of those deaths are necessarily due to H1N1, the percentage of influenza positive tests among the ILI patients keeps rising and stands at 37.5% during week 41 [20]. Pediatric mortality from H1N1 already exceeds annual counts from previous years. 

In the current H1N1 influenza pandemic, antiviral drugs are actively prescribed to symptomatic individuals in some countries [21],[9]. Under the circumstances of elevated mortality and hospitalization rates in the US, with a high proportion of ILI patients actually having H1N1, we think it is worthwhile to consider a revision to the current antiviral usage recommendations that will encourage physicians to prescribe antivirals at their discretion to individuals with moderate to severe symptoms who show up for treatment.

**The cons of antiviral usage:** Having so far discussed the pros of antiviral usage, we shall now list some of its potential cons.

Antiviral resistance:  Oseltamivir resistance levels in H1N1 strains in the US are currently low, standing at 0.5% during week 41 [20]; those levels have not increased in recent weeks. Resistance levels are even lower (0.17%) in the UK [22], where more extensive antiviral usage takes place, with a higher proportion of the infected individuals being treated with antiviral drugs.  Given the fact that sizeable antiviral resistance has not emerged so far in the UK with higher treatment levels than in the US, we think it is unlikely that if the US treatment levels increase to match the UK ones, significant levels of resistance will emerge in the upcoming weeks before large quantities of vaccine become available.  Moreover, prior mathematical modeling studies [23],[24] have shown that if the epidemic is already large (as it is now) at the time that large-scale antiviral treatment begins, then resistant strains will be unlikely to ascend to high frequency in the population before the epidemic is over, even in the absence of vaccination.

Stockpile depletion:  It seems likely that a small percentage of infected persons have sufficiently serious symptoms to seek care. An estimate from New Zealand [25] is that only one out of 18 did, although survey data in the same population suggested that the proportion seeking care was closer to 25% (MG Baker, personal communication). US data suggest a similar range of uncertainty, with self-reports of 42-58% of persons with ILI seeking care [26], but comparisons of ILI to physician visits suggest much lower numbers [27],[28]. In Chile, 650,000 antiviral courses were dispensed in a population of 17 million, representing coverage of 3.8% of the population. Such demand can be readily met with the US stockpile, unless demand levels increase sharply.

Excessive demands on health care provision:  This phenomenon may be exacerbated by availability of more antivirals and cannot be fully predicted. We note, however, that more pro-active antiviral distribution policies did not overwhelm healthcare systems neither in Chile nor in the UK.

Adverse effects of antiviral treatment: Some adverse effects to antiviral treatment exist [29] and should be discussed between doctors and patients. Those effects should be weighted against alleviation of symptoms and decreasing the risk of deterioration into a severe condition.

Cost: The cost of more extensive use of antivirals would fall to a number of parties, including federal and state governments (who would incur costs in replenishing stockpiles drawn down for treatment), and individuals and insurance carriers, who would pay for privately obtained courses.   These costs should be weighed against the potential health benefits, described above, and economic benefits of reduced symptom duration, obtained from more aggressive treatment.

**Funding Information:** Research is supported by the Center of Excellence award from MIDAS.

**Competing interests:** ML discloses consulting fees from the Avian/Pandemic Flu Registry (Outcome Sciences), funded in part by Roche. EG declares no competing interests. 

**Acknowledgement**: We thank Leon Danon and Martin Lajous for help in preparation of this manuscript.

## References

[ref-3941625633] Aoki FY, Macleod MD, Paggiaro P, Carewicz O, El Sawy A, Wat C, Griffiths M, Waalberg E, Ward P, IMPACT Study Group: Early administration of oral oseltamivir increases the benefits of influenza treatment. J Antimicrob. Chemother. 2003; 51(1) 123-9http://www.ncbi.nlm.nih.gov/pubmed/12493796 10.1093/jac/dkg007 12493796

[ref-3355735356] Burch J, Corbett M, Stock C, Nicholson K, Elliot AJ, Duffy S, Westwood M, Palmer S, Stewart L: Prescription of anti-influenza drugs for healthy adults: a systematic review and meta-analysis. Lancet Infect Dis. 2009 Aug 7. [Epub ahead of print]http://www.thelancet.com/journals/laninf/article/PIIS1473-3099(09)70199-9/abstract 19665930 10.1016/S1473-3099(09)70199-9

[ref-70917561] McClellan K, Perry CM, Oseltamivir: a review of its use in influenza. Drugs. 2001;61(2):263-83.http://www.ncbi.nlm.nih.gov/pubmed/11270942 10.2165/00003495-200161020-00011 11270942

[ref-34601101] Goldstein E, Cowling B, O’Hagan J, Danon L, Fang V, Hagy A, Miller JC, Reshef D, Robins J, Biedrzycki P, Lipsitch M. Oseltamivir for treatment and prevention of pandemic influenza A/H1N1 virus infection in households, Milwaukee, 2009, Submitted. 10.1186/1471-2334-10-211 PMC2919545 20642862

[ref-1896202309] Halloran, EM, Hayden FG, Yang Y, Longini IM, Monto AS: Antiviral Effects on Influenza Viral Transmission and Pathogenicity: Observations from Household-based Trials. Amer. J Epidem. 2007 165(2):212-221http://www.ncbi.nlm.nih.gov/pubmed/17088311 10.1093/aje/kwj362 17088311

[ref-3533573873] Ng S, Cowling BJ, Fang VJ, Chan KH, Ip DKM, Cheng CKY, Uyeki TM, Houck PM, Peiris JSM, Leung GM. Effects of oseltamivir treatment on duration of clinical illness and viral shedding, and household transmission of influenza virus. Clinical infectious Diseases, 2009 (in press).http://www.themacraegroup.com/2009-symposia/xi-international-symposium-on-respiratory-viral-infections/poster-abstracts/effects-of-oseltamivir-treatment-on-duration-of-symptoms-and-viral-shedding-and-household-transmission-of-influenza-virus 10.1086/650458 PMC2840043 20121573

[ref-1027733278] Kaiser L, Wat C, Mills T, Mahoney P, Ward P, Hayden F. Impact of oseltamivir treatment on influenza-related lower respiratory tract complications and hospitalizations. Arch Intern Med. 2003 Jul 28;163(14):1667-72.http://www.ncbi.nlm.nih.gov/pubmed/12885681 10.1001/archinte.163.14.1667 12885681

[ref-3920185087] Jain S, Kamimoto L, Bramley AM, Schmitz AM, Benoit SR, Louie J, et al. Hospitalized Patients with 2009 H1N1 Influenza in the United States, April-June 2009. N Engl J Med 2009 Oct 8.http://content.nejm.org/cgi/content/full/NEJMoa0906695 10.1056/NEJMoa0906695 19815859

[ref-6217480] Ministerio de Salud, Chile. Influenza pandemica (H1N1) 2009 , FECHA: 19/08/09http://www.redsalud.gov.cl/portal/url/item/7233bd763811db01e04001011f0115af.pdf

[ref-2983368355] Ministerio de Salud, Chile. Influenza pandemica (H1N1) 2009. REPORTE 21 DE OCTUBRE DEhttp://www.redsalud.gov.cl/minsalaudios/reporte22octubre.pdf

[ref-3581055002] Ministerio de Salud de la Nacion, Republica Argentina. Influenza pandemica (H1N1) 2009.http://www.iom.edu/~/media/Files/Activity%20Files/PublicHealth/MicrobialThreats/H1N1%20Pandemic%20SEP15/Uez%20WEB.ashx

[ref-3316853877] Ministerio de Salud de la Nacion, Republica Argentina. Influenza pandemica (H1N1) 2009.http://www.msal.gov.ar/archivos/Informe_SE_39-_ARG_COM%5B1%5D.pdf

[ref-4000953354] Goldstein E, Miller JC, O’Hagan J, Lipsitch M. Predispensing of Antivirals to High-Risk Individuals in an Influenza Pandemic. PLOS Currents Influenza. Submitted.http://knol.google.com/k/edward-goldstein/predispensing-of-antivirals-to-high/1uji2pldf66z5/1# 10.1371/currents.RRN1007 PMC2762331 20029604

[ref-1950910876] CDC. Updated Interim Recommendations for the Use of Antiviral Medications in the Treatment and Prevention of Influenza for the 2009-2010 Season.http://www.cdc.gov/h1n1flu/recommendations.htm

[ref-3256064730] CNN. Oct. 13, 2009 Study: Underlying conditions playing less of role in H1N1http://www.cnn.com/2009/HEALTH/10/13/h1n1.preexisting.conditions/index.html

[ref-2328609532] Perez-Padilla R, de la Rosa-Zamboni D, Ponce de Leon S, Hernandez M, Quinones-Falconi F, Bautista E, et al. Pneumonia and respiratory failure from swine-origin influenza A (H1N1) in Mexico. N Engl J Med2009 Aug 13;361(7):680-9.http://content.nejm.org/cgi/content/full/NEJMoa0904252 10.1056/NEJMoa0904252 19564631

[ref-3809715517] Rello J, Rodriguez A, Ibanez P, Socias L, Cebrian J, Marques A, et al. Intensive care adult patients with severe respiratory failure caused by Influenza A (H1N1)v in Spain. Crit Care2009 Sep 11;13(5):R148.http://www.ncbi.nlm.nih.gov/pubmed/19747383 10.1186/cc8044 PMC2784367 19747383

[ref-3881617744] H1N1 leading to increased flu rates, more pediatric deaths.http://www.rwjf.org/publichealth/digest.jsp?id=23828

[ref-3968686575] CBS News. Emotional Plea from H1N1 Victim's Parents. Sep. 30, 2009http://www.cbsnews.com/stories/2009/09/30/earlyshow/health/main5352499.shtml

[ref-1169801891] CDC. A weekly influenza surveillance report prepared by the influenza divisionhttp://www.cdc.gov/flu/weekly/

[ref-1583840560] Health Protection Agency (UK). 26 October, 2009. Summary of prescribing guidance for the treatment and prophylaxis of influenza like illness.http://www.hpa.org.uk/web/HPAwebFile/HPAweb_C/1243581475043

[ref-4290296358] Weekly pandemic flu media update. Health Protection Agency. 22 October 2009http://www.hpa.org.uk/web/HPAwebFile/HPAweb_C/1254510639308

[ref-822877241] Lipsitch M, Cohen T, Murray M, Levin BR (2007) Antiviral Resistance and the Control of Pandemic Influenza. PLoS Med 4(1): e15.http://www.plosmedicine.org/article/info:doi/10.1371/journal.pmed.0040015 10.1371/journal.pmed.0040015 PMC1779817 17253900

[ref-3641836168] Wu JT, Leung GM, Lipsitch M, Cooper BS, Riley S (2009) Hedging against Antiviral Resistance during the Next Influenza Pandemic Using Small Stockpiles of an Alternative Chemotherapy. PLoS Med 6(5): e1000085.http://www.plosmedicine.org/article/info:doi%2F10.1371%2Fjournal.pmed.1000085 10.1371/journal.pmed.1000085 PMC2680070 19440354

[ref-1267959064] Baker MG, Wilson N, Huang QS, Paine S, Lopez L, Bandaranayake D, Tobias M, Mason K, Mackereth GF, Jacobs M, Thornley C, Roberts S, McArthur C. Pandemic influenza A(H1N1)v in New Zealand: the experience from April to August 2009. Euro Surveill. 2009;14(34):pii=19319.http://www.eurosurveillance.org/ViewArticle.aspx?ArticleId=19319 10.2807/ese.14.34.19319-en 19712648

[ref-1597787599] Reed C, Angulo FJ, Swerdlow DL, Lipsitch M, Meltzer MI, Jernigan D, et al. Estimates of the prevalence of pandemic (H1N1) 2009, United States, April–July 2009. Emerg Infect Dis [serial on the Internet] 2009 Dechttp://www.cdc.gov/EID/content/15/12/pdfs/09-1413.pdf 10.3201/eid1512.091413 PMC3375879 19961687

[ref-526394904] Presanis AM, Lipsitch M: The severity of pandemic H1N1 influenza in the United States, April – July 2009http://knol.google.com/k/anne-m-presanis/the-severity-of-pandemic-h1n1-influenza/agr0htar1u6r/16# 10.1371/currents.RRN1042 PMC2762775 20029614

[ref-1960029376] Metzger KB, Hajat A, Crawford M, Mostashari F (2004) How many illnesses does one emergency department visit represent? Using a population-based telephone survey to estimate the syndromic multiplier. MMWR Morb Mortal Wkly Rep 53 Suppl: 106-111http://www.ncbi.nlm.nih.gov/pubmed/15714638 15714638

[ref-3566032739] CDC. Antiviral Agents for Seasonal Influenza: Side Effects and Adverse reactions.http://www.cdc.gov/flu/professionals/antivirals/side-effects.htm

